# Salient stakeholders: Using the salience stakeholder model to assess stakeholders’ influence in healthcare priority setting

**DOI:** 10.1016/j.hpopen.2021.100048

**Published:** 2021-07-17

**Authors:** Lydia Kapiriri, Shaghayegh Donya Razavi

**Affiliations:** Department of Health, Aging and Society, McMaster University, 1280 Main Street West Hamilton, Ontario, Canada

**Keywords:** Stakeholder analysis, Salience stakeholder analysis framework, Priority setting

## Abstract

Stakeholders play an important role in health priority setting, and their roles have been discussed in the literature, mainly in relationship to their power. An emerging body of literature is focusing on the legitimacy of the stakeholders. Using the case of the Uganda health system, the overall aim of this paper is to assess the utility of the salience stakeholder analysis framework in identifying the most salient stakeholders in health-care priority setting.

**Methods:**

This was a qualitative case study involving 57 key informant interviews with national and district level policy makers and a review of policy documents. Interview data were analyzed using QSR NVivo10 qualitative data analysis software. Analysis was guided by the salience stakeholder analysis framework.

**Findings:**

Among the eight groups of stakeholders identified by the respondents, the politicians were found to be the most salient stakeholders. However, stakeholders’ salience varied depending on the type of decision, the nature of health issue and how and who tabled the health issue.

**Conclusion:**

The salience stakeholder analysis framework, originating from the business management and political science disciplines, provided a more comprehensive stakeholder analysis by supporting the concurrent consideration of power, legitimacy and urgency in stakeholder analysis for health care priority setting.

## Highlights

• Stakeholder (SH) power and legitimacy affect healthcare priority setting (PS).
• Health policy stakeholder analysis frameworks (FW) mainly assess stakeholder power.
• What is the contribution of a business management and political science FWs?
• Salience stakeholder analysis (SSA) FW assesses SH power, legitimacy, urgency.
• The SSA framework supported comprehensive SH analysis in health care PS.

## 1 Introduction

There are strong arguments for involving a wide range of stakeholders in healthcare priority setting. Priority setting, in this context, refers to the ordering of potential health system interventions for the purposes of resource allocation. First, priority setting is thought to be value-laden and there is a tendency for the decisions to reflect the values of the people involved or represented in the process. Hence, involving a wide range of stakeholders in the prioritization process facilitates the consideration of a broad range of values [1], [2], [3]. Second, contrary to the perception that priority setting decisions are technical, in practice priority setting decisions are both political and value-laden. Hence, involving a varying range of stakeholders enhances accountability and the legitimacy of the priority setting decisions [1], [4], [5], [6], [7], [8]. In relationship to the politics of priority setting, involving a wide range of stakeholders creates shared decision-making on key policy issues, which supports consensus building around critical decisions and contributes to the resolving of the moral conflicts that are pervasive in pluralistic societies [9], [10]. Lastly, stakeholders participation improves the quality and acceptability of the prioritization decisions, and provides a broad range of perspectives that are necessary in policy-making [11], [12], [13], [14], [15].

However, involving a wide range of stakeholders in decision-making may also present some challenges. Stakeholder engagement can be costly, time consuming [1], and if done poorly, stakeholder engagement may have detrimental results [1], [16], [17]. For example, since stakeholders have different interests, it is not uncommon to find that they disagree on decisions. In such instances, if consensus is the aim, it may take time to arrive at a decision. Furthermore, if stakeholders’ inputs are not reflected in the decisions that are made, the participants may perceive their contribution as tokenisms, which can hamper future engagement [18], [19]. Lastly, it is not uncommon to find that participatory decision-making processes, that are not well facilitated to ensure that all stakeholders have an equal chance to participate, are filled with power imbalances whereby “powerful” stakeholders dominate and overly influence the processes [20], [21], [22]. Stakeholder analysis is an important step in understanding the nature of this influence and mitigating the power imbalances.

There is a growing body of literature on stakeholder participation in health care priority setting. This literature has mainly focused on assessing which stakeholders commonly involved in or excluded from priority setting [1], [23], and the degree to which the different stakeholders actually participate in health care priority setting [1], [20], [24]. Another body of literature has focused on analysing the roles and leverages of the various stakeholders; as well as the legitimacy of the various stakeholders [20], [22].

A limited body of literature has focused on frameworks used in stakeholder mapping and analysis. Stakeholder mapping involves identifying the facilitators to and inhibitors of stakeholder participation [25], [26], [27].

The literature to stakeholder involvement and stakeholder mapping and their legitimacy is relevant to health care priority setting. However, there is limited literature that that pulls all these relevant aspects of stakeholder participation within the context of healthcare prioritization. Furthermore, this literature has not focused on identifying the legitimate, yet critical stakeholders, who might have the greatest influence, and should hence be “courted” when setting healthcare priorities. Frameworks from other disciplines could be of potential use to healthcare priority setting researchers.

The business and political science literature discusses frameworks and criteria that could be useful when identifying the critical stakeholder(s) in healthcare priority setting. For example, Mainardes, Alves & Raposo (2012) summarize some of the frameworks and criteria that can be used to identify and classify the important stakeholders under the following categories: stakeholder power and level of interest; strategic and moral stakeholders; potential powers; primary versus secondary; network density and centrality of organization focus; classical, stake watchers and stake keepers; power of influence; and impact and affinity [27]. Furthermore, frameworks such as the Power Versus Interest Grid (2X2 matrix) [28], the Onion Model [29], the ‘9C’s stakeholder analysis [30], the Force Field analysis [31], the Advocacy Coalition Framework [32], and Elster’s framework [25]; all endeavour to analyse stakeholders’ levels of power and influence. While these frameworks would enable us to identify the critical stakeholders in healthcare priority setting, they do not address stakeholder issues related to their legitimacy and urgency (Table 1).

**Table 1:** Summary of stakeholder analysis framework mapping frameworks.

Model/Analytical Framework	Summary features
The Power Versus Interest Grid	The goal is to map stakeholder importance, influence, interest and yield. Maps power on one axis and interest in the other to identify the facilitators, sideliners, beneficiaries, and key for business stakeholders
The Onion Model	Identifies the primary, secondary and contextual stakeholders ranging from the core team, to the external stakeholders
The ‘9C’ stakeholder analysis	Identifies all organizational stakeholders: the champions, contributors, commissioners, customers, collaborators, commentators, consumers, channels and competitors
Advocacy coalition framework (ACF)	Explains stakeholder behavior and policy outcomes over long periods of time by focusing on the interaction of advocacy coalitions-each consisting of actors/stakeholders from a variety of institutions-within a policy subsystem.
Elster’s framework	Identifies relevant actors and categorizes them by their roles, identifies their concerns, and their potential leverages in a decision-making process.
The force field analysis	Maps out the project progress identifying any forces that might be of influence e.g. the reactions, restraining forces, and difficulties. Identifies the stakeholder who might be driving or constraining the project
The Salience stakeholder model	The goal is to define the most important stakeholder by mapping stakeholders according to their power, urgency and legitimacy (Table 2)

A framework that supports the analysis of the stakeholders’ power while recognizing their legitimacy and urgency (all of which are relevant in healthcare prioritization), such as the *salience stakeholder analysis* framework, may provide a more comprehensive analysis.

The *salience stakeholder analysis framework (*analysing stakeholders’ legitimacy, power, and urgency) was developed and has been used by political science and business management researchers to identify salient stakeholders.

Mitchell, Agle, and Wood’s (1997) *salience stakeholder analysis* framework maps out stakeholders according to stakeholder attributes namely, legitimacy, power, and urgency [33]. The framework conceptualizes: (i) *Legitimacy* as the general perception that the actions of a given entity are desirable, appropriate, and acceptable within a socially constructed context; (ii) *Power* as a relationship wherein one actor can influence the actions of another actor, specifically to do something they otherwise would not do, and (iii) *Urgency* as the degree to which stakeholder claims require immediate attention (can be based on either time sensitivity or criticality). Lastly, salience is defined as the degree to which managers prioritize the competing stakeholder claims. Based on these attributes, Mitchell, Agle, and Wood’s (1997) developed a typology that categorizes stakeholders based on their having one, two, or all of these attributes to identify the potential latent, expectant, and definitive stakeholders (see Table 2).

**Table 2:** Stakeholder typologies in the salience stakeholder model.

Stakeholder class	Typology	Attributes
Latent	Dormant	Power
	Discretionary	Legitimacy
	Demanding	Urgency
Expectant	Dominant	Power & Legitimacy
	Dangerous	Power & Urgency
	Dependent	Legitimacy & Urgency
Definitive	Definitive	Power, Legitimacy, Urgency

*Latent Stakeholders* possess only one of the three attributes; hence they have low salience and are less likely to have meaningful influence on the decision-making processes. A latent stakeholder that possesses only power, is considered *dormant*, one with only legitimacy is considered *discretionary,* while one with only urgency is *demanding*. A dormant stakeholder has the power to impose their will but lacks the legitimacy and urgency. Similarly, a discretionary stakeholder lacks the power and urgency, while the demanding stakeholder lacks both power and legitimacy. Demanding stakeholders can be considered “noise-makers” since they lack the power or legitimacy to move their claims to a more salient position. Any latent stakeholder can become more salient by acquiring any one or both of their missing attributes.

*Expectant stakeholders* possess two of the attributes. According the framework, expectant stakeholders include dominant, dependant, and dangerous stakeholders. *Dominant* stakeholders have both power and legitimacy. These may influence decision-making since they are perceived to be legitimate and are able to act. *Dependent* stakeholders have legitimacy and urgency. Their lack of power means that they will rely on the stakeholders with the power to represent their interests. Lastly, *dangerous* stakeholders possess both power and urgency. Dangerous stakeholders’ influence maybe perceived as coercive since they lack legitimacy.

*Definitive stakeholders* possess all three attributes and are hence the most salient.

The framework’s ability analyse stakeholders’ urgency and legitimacy makes it potentially useful in facilitating health system researchers’ assessment of the salience of the different stakeholders who are involved healthcare priority setting. This paper contributes to the political science and health systems literature by testing the utility of this framework in healthcare prioritization.

## 2 Overall goal and objectives

The overall aim of this paper is to assess the utility of the *salience stakeholder analysis framework* in identifying the most salient stakeholders in healthcare priority setting.

**Specific objectives are:**

1. To identify and analyse the roles played by the various stakeholders in national-level healthcare priority setting in Uganda.
2. To apply *the salience stakeholder analysis framework* to identify the most critical stakeholders in healthcare prioritization.
3. To discuss the contributions of *the salience stakeholder analysis framework* to the literature and debate on stakeholder participation in healthcare priority setting.

## 3 Methods

This was a qualitative case study involving key informant interviews and a review of policy documents.

*Study setting and study population*: The study was conducted in Uganda between 2013 and 2015; followed with a validation of the findings in 2017. We interviewed policymakers at the national and district levels and reviewed policy documents at both levels. The Ugandan health system structure and healthcare prioritization processes and challenges have been described in the literature [2], [8], [19], [22], [23], [24]. Summarily, Uganda has a decentralized health care system whereby the national level decision-makers are responsible for policy direction, and monitoring and evaluation, while district level decision-makers are responsible for policy implementation [8], [24]. The national and district level decision-makers are involved in priority setting at their respective levels [22], [23], [24]. Similar to other LICs, the limited budget allocations to the health sector has led to the involvement of several (non-state) stakeholders in the national healthcare prioritization processes. Unfortunately, these stakeholders’ priorities may not always align with the national priorities, and their legitimacy has been questioned [8], [19], [24].

*Sampling*: A mixture of purposeful and snowball sampling was used to identify the relevant respondents. Respondents were identified by virtue of their involvement in national and district level priority setting. Index respondents at the national level were identified through the Ministry of Health webpage; based on the six indicator cases, these were program leads. After interviewing the index respondents, they were requested to identify additional relevant respondents. The district level interviews involved purposeful selection of three districts from three of the five regions in Uganda (to reflect variations in: economic status, rurality, and duration since decentralization). Within each district, we purposefully identified and interviewed all members of the district health team who are involved in district level priority setting.

*Data collection*: This paper reports findings from a larger qualitative study whose purpose was to describe and evaluate national and district level priority setting in Uganda. Data was collected by a trained research assistant, using a pilot tested interview guide. Respondents were asked about the stakeholders involved in priority setting; specific questions included: i) *which stakeholders are involved in priority setting?* (ii) *What roles do the stakeholders hold?* (ii) *How do these stakeholders influence healthcare priority setting?*

*Data analysis*: Interviews were recorded with permission from the respondents. All interviews were transcribed verbatim and analysed using QSR NVivo10 qualitative data analysis software. The initial micro-coding involved identifying texts that were related to stakeholders and these were labeled stakeholders. Secondary coding involved categorizing the stakeholder texts along the various dimensions which were again labeled. Related categories were grouped together into themes to provide a rich description of the various stakeholders identified in the study. This included information on stakeholder names, stakeholder roles, legitimacy, urgency power and influence. Based on these themes, further analysis involved using the *salience stakeholder analysis* framework (Table 1, Table 2) to identify the most salient stakeholder(s). The framework’s attributes were applied to the stakeholders’ roles and influence, as described by the respondents to identify the most salient stakeholder(s). This involved three stages. Since the identified stakeholder roles and influence were aligned with the three attributes (*Power, legitimacy and Urgency*) in the analysis framework, the first stage involved identifying which stakeholder description identified only one of the three attributes. Depending on the attribute, the corresponding typology was identified. All stakeholders with *one attribute* fell under the *latent* stakeholder class. Second, we assessed which stakeholder descriptions aligned with a combination of two attributes of the three attributes. Depending on the attribute combination, the stakeholders were allocated the corresponding typology. All stakeholders with *two attributes* fell under the *expectant* stakeholder class. Lastly we identified stakeholders whose description covered all *three* attributes; these fell under the definitive typology and *definitive* stakeholder class.

## 4 Results

We interviewed a total of 57 respondents (38 at the national and 19 at the district level). Respondents included Ministry of Health (MOH) officials (policymakers), development assistance partners (DAPs), non-governmental organizations (NGOs), civil society organizations (CSOs), and district officers (DO) (Table 3).

**Table 3:** Description of the study participants.

Level of priority setting	Type of respondents	Total
National (38)	• Ministry of Health (MOH) officials (policymakers)	48
	• Development assistance partners (DAPs)	5
	• Non-governmental organizations (NGOs)	2
	• Academics	2
District level (19)	• District planners and Politicians	12
	• Members of the District Health teams	15
Total		57

The respondents identified the various stakeholders involved in healthcare prioritization including Ministry of Health officials (policymakers), development assistance partners, non-governmental organizations, civil society organizations, district officers, media and academics. Most of the identified stakeholders reflected the study respondents’ designations

According to our respondents, the *Ministry of Health officers* (MOH) have the legal mandate and the primary responsibility for national level healthcare priority setting, as illustrated by a respondent:

*“…most of the interventions that are implemented by the organizations come as a result of priorities set by Ministry of Health whereby requests for proposals (RSA) are put in the (News) papers and then organizations write proposals responding to the RSA put in the papers and then implement those projects in relation to the Ministry of Health priorities.”* (NGO_3)

National-level priority setting also involves stakeholders such as *Development Assistance Partners (DAPs), Non-government organizations (NGOs), politicians, academics*, and sometimes *Civil Society Organizations (CSOs)*. Some of the DAPs, and NGOs, play an important role by providing resources (both financial and technical expertise) that support the prioritization process, and/or the implementation of the identified priorities. The politicians and more specifically, the members of parliament, were reported to be responsible for allocating financial resources across the different sectors (including the health sector), at the national level. Furthermore, respondents reported that politicians and civil society organizations play an additional role as public representative and advocates. As explained by a respondent:

*“…the other important one (group) is Parliament because Parliament reviews the budgets, they look at performance, they make decisions, they own policy even if policy originates from the Ministry of Health that policy is perceived to come from the Parliament, it is the policy making organ. They are representing the community…”* (CSO_4)

Furthermore, *the media* was identified as playing the role of knowledge brokers, communicating information and perspectives about healthcare priorities to the public. They also publish the challenges related to health program implementation.

## 5 Mapping the above stakeholders to the *salience stakeholder framework* attributes

This section presents further analysis of the stakeholders’ salience based on the analytical framework, since the stakeholders did not neatly fit under a single classification, the interactions between the stakeholder categories are the main focus of our discussion. The section is organized according to the stakeholder classes presented in Table 1, namely: the latent, expectant, and definitive stakeholders. For each stakeholder class, we discuss their respective typologies and attributes.

### 5.1 Latent stakeholders

According to the framework, latent stakeholders are either *dormant, discretionary,* or *demanding*. Study respondents described several groups of stakeholders that could be categorizes as either dormant or discretionary. However, no stakeholders could be categorised as demanding.

### 5.2 Dormant stakeholders

*Development assistance partners* were predominantly described as having the power to influence priority setting. This was by virtue of DAPs having the financial and technical expertise to support the prioritization process and the implementation of the priorities, as expressed by a respondent:

*“…What I’m trying to say is that the man with the pocket is the man who plays the bigger bit in prioritization…So resource mobilization is a very big problem and it's a big challenge to setting priorities. And many times, as we set priorities, we do not set our own priorities, we end up looking at what the donors’ priorities are and then we begin to get ourselves around that.”* (NGO_2)

The DAP’s *power* can be understood in terms of either utilitarian power (which involves the provision of material resources or other incentives), or coercive power (where they can exert their influence either through “force” or through “threats”). In regard to the utilitarian power, as expressed in the quote above, DAPs provide resources. When discussing the “coercive power”, respondents identified instances whereby DAPs give conditions on how the DAP funds should be used. This can be problematic when the DAP priorities are not aligned with the locally identified priorities, and there is lack of flexibility, on the DAPs part, to allow the resources to be used on the locally identified priorities as demonstrated in the quote below.

*“…We [local governments] tell them [DAPs] we want to fund (for example) neglected diseases you know …the people suffer in the northern area, they (DAPs) say no my money … is for HIV/AIDS (for example)…”* (MOH_ 1)

### 5.3 Discretionary stakeholders

*District officers*, *academics, and the media* fulfilled the typology of discretionary stakeholder, who seemed to possess only legitimacy and to lack the power and urgency, according to our respondents. The legitimacy of the district officers stemmed from their understanding of the issues within their local contexts and as implementers of the identified priorities.

*“But normally consultation about priorities that should be addressed is supposed to be a bottom-up from what we call the frontline guys, the District Medical Officers. You start discussing what are the issues in district A, B, C, D, and then it comes up…”* (DAP _2)

However, respondents from the district reported a lack of power to influence national level priority setting, despite the decentralization policies that devolved decision-making powers from the central to local governments, as demonstrated by the following respondent:

*“We need to set our own priorities according to the local context, and local needs. We would be doing very well if we did… So the issue of decentralization also needs to be considered and the district be empowered more, so that they are able to implement their priorities.”* (DO_ 2)

The academics were perceived as legitimate by virtue of their ability to collect and share research evidence. However, although district officers, the academics, and media were perceived as legitimate, they lacked urgency and the power to influence priority setting.

### 5.4 Demanding stakeholders

No stakeholders fulfill the described attributes, which was surprising.

### 5.5 Expectant stakeholder

The expectant stakeholder class includes stakeholders who are either *dominant, dependent,* or *dangerous*.

### 5.6 Dominant stakeholders

Dominant stakeholders possess both the power and legitimacy to influence priority setting. According to the respondents’ description of the stakeholders, several could fit under this category. However, stakeholders that could consistently fit under this category are the *politicians* and the *Ministry of Health officials*. As discussed above, politicians control the national budget and represent the public’s interests. Several respondents deemed politicians legitimate since they are elected public representatives;

*“…Like, when politicians go to their communities, the communities tell them where their concerns. And depending on how often the concern comes up, it can be prioritised…”* (MOH_2)

However, while the MOH officials were perceived as dominant stakeholder, there was also a perception that when there is weak leadership and limited resources, the MOH’s role as dominant stakeholders is undermined by, for example, the DAPs who have the resources (and hence power-discussed above).

*“…over time the Ministry leadership has somehow weakened so the consultative process has died down. So, the process has now been restricted to a few individuals and the donors taking the upper hand…”* (MOH_20)

### 5.7 Dependent

Dependent stakeholders possess both legitimacy and urgency. The public, civil society organizations, and NGOs could be categorised as dependent stakeholders. The legitimacy of these stakeholder groups stems from their role as representing the general public’s interests in priority setting. By virtue of representing the public and perceiving the public as the group that stands to be impacted by the priority setting decisions, all the stakeholders in these groups have some degree of urgency and legitimacy.

### 5.8 Dangerous

Dangerous stakeholders possess both the power and urgency. While the respondents did not describe a stakeholder who consistently fulfilled both attributes of dangerous stakeholders, several stakeholders seemed to move in and out of this category. For example, around the time of elections, politicians were perceived as having a tendency to influence priority setting to respond to the wants of the populations because of the politicians’ desire to be re-elected. Although they are perceived as legitimate, the actions taken to use their power and to create an urgency (due to their urgent need for re-election) were perceived as illegitimate. Furthermore, some respondents identified characteristics of dangerous stakeholders when talking about DAPs, whereby they described instances when a DAP identifies a priority, which they (independent of the local institutions) deem urgent, and use the fact that they possess the necessary resources as a source of power and leverage to implement their (DAP) agendas.

*“I’ll give an example like we, within the priorities that we are set what we had agreed was that a cost-effective study was done. Now somebody comes up and gives a donation and says now you can take it on and start vaccinating … Now with that development, now the priorities seem to change…”* (MOH_30)

## 6 Definitive stakeholder

Definitive stakeholders possess all the three attributes: the power, legitimacy and urgency. While it was difficult to identify a group of stakeholders who were consistently perceived as a definitive stakeholder, one, rather controversial stakeholder, who seems to fulfill all the attributes of a definitive stakeholder were the politicians. More specifically, the president (and first lady) were identified by several respondents as possessing both the power and urgency to enact and rally behind some health priorities. As elected political leaders, they were perceived by some, as legitimate. For example, respondents narrated instances whereby the president has prioritised a health issue e.g. HIV/AIDS, maternal newborn and child health (MNCH), and vaccination and these have been automatically prioritized by the MOH. Sometimes, their priorities are aligned with their election mandate, and hence have an element of urgency (as discussed above). More explicitly in 2005, the President is reported to have convened a meeting on the state of MNCH where he tasked the Ministry of Health to develop a master plan to address the issue of high maternal mortality. Consequently, the MOH along with other sectors and partners renewed their commitment to address maternal health issues and developed the “Roadmap for Accelerating the Reduction of Maternal and Neonatal Mortality and Morbidity in Uganda” [34].

## 7 Discussion

This study presents findings from a qualitative study assessing the salience of the various stakeholders involved in healthcare priority setting using a framework which was developed and has been used in political science and business management. The analytical strength of this framework lies within its ability to assess the power, legitimacy, and urgency of the stakeholders to facilitate the identification of the most important (or salient) stakeholder. According to this framework, the most important stakeholder should be “courted” if a project/program is to be successful [33]. This paper contributes to the previous literature that has used this framework by basing the analysis on empirical data from the stakeholders themselves. It is also the first paper, to the best of our knowledge, that applies this framework to healthcare decision making.

The framework proved to be very relevant to the health sector and priority setting, in particular, since it catered for legitimacy; an attribute that was very important to the respondents. Therefore, while the other frameworks would have been appropriate for identifying the most influential stakeholders [25], [27], [28], [29], [30], [31], [32]; they lacked the ability to assess an attribute that was relevant to the respondents.

The salience stakeholder framework enabled us to identify the salient stakeholders as politicians. While the literature on the role of politicians’ role in priority setting is divided with regards to their legitimacy [35], [36], [37], the literature that supports the role of politicians qualifies this by emphasizing that they could have an important role when thought to be acting in the public’s and not their own selfish interests (i.e. to be re-elected) [23], [38]. Politicians’ legitimacy comes, in part, from their being elected representatives of the public [39]. Hence, although the public may not always be involved in priority setting [22], [40], they may play a critical indirect role through their influence on the politicians [8], [22].

The findings that politicians, and not DAPs, were the most salient stakeholders was surprising and contrary to the literature on stakeholders’ influence over LIC health systems [22], [41], [42], [43]. This could be explained by the salience stakeholder framework which we used, which included legitimacy as an important attribute in stakeholder analysis [33]. Hence, although DAPs possessed the power (which is emphasized in other stakeholder analysis models [27]) their perceived lack of legitimacy and urgency made them latent as opposed to salient stakeholders. However, this categorization was with exceptions; DAPs whose actions were aligned with the national priorities were considered legitimate. Furthermore, DAPs who operate under the principles of equity and social justice to reduce the unfair disease burden in low-income countries may have a claim to the urgency attribute [42], [43]. Hence, would a DAP who uses their power to influence the prioritization and implementation of national health issues that affect the most vulnerable be deemed salient? This makes the DAPs and their actions difficult to be placed in a specific category.

The public, CSOs, NGOs, district officers, and MOH had moderate salience since they collectively possessed two attributes (legitimacy and urgency). While the technical officers in the district and MOH were deemed legitimate due to their technical expertise, and are often believed to act in the public’s interest [41], their limited access to resources hamper their ability, and hence, effective power to influence healthcare priority setting. Based on the salience model, it may be logical to propose that the most salient stakeholders should be the technocrats if they use their technical knowledge, are acting in the interests of the public and are provided with the resources that are necessary to ensure that the set priorities are implemented.

While the framework identified the salient stakeholders, high quality priority setting should be participatory where all relevant stakeholders play an important role. Emphasizing and courting the salient stakeholders may further marginalize stakeholders that lack power. Democratic and equitable stakeholder participation in priority setting would require dealing with the power imbalances that might arise in the process of courting the salient stakeholders.

## 8 Study limitations

Similar to all qualitative studies, we cannot generalize the findings. However, since we interviewed respondents at two decision making levels, and interviewed respondents who are actually involved in the prioritization process; the findings are still relevant to this and other similar contexts.

There were limitations with the framework’s application to healthcare prioritization. First, it was difficult to fit the stakeholders in one explicit class; second, within the context of healthcare priority setting, stakeholder salience may vary according to the decision being made. Third, “courting” of salient stakeholders may further the power imbalances in stakeholder influence.

## 9 Conclusion

This study demonstrated the utility of a business management and political science model (the salience stakeholder model) in identifying the highly salient stakeholder(s) in national healthcare priority setting. The Salience Stakeholder framework, by emphasizing legitimacy and urgency, led to the identification of politicians as the most salient stakeholders. However, the salience of the various stakeholders may vary depending on the type of decision, the timing of the decision, and nature of and how the health issue under question arose and who is affected by the health problem.

While the previous literature on stakeholder engagement focused on either stakeholder power; this framework provided the opportunity to concurrently assess three attributes (power, legitimacy and urgency) of stakeholder participation in healthcare priority setting. The application of the framework revealed that rather than stakeholders being strictly categorised in one class of salience as depicted by the framework, stakeholders tended to move from one class to the other. Hence, while healthcare decision-makers should consider using this framework in their stakeholder mapping, the mapping and analysis should be done for every priority setting decision, since the stakeholder roles and level of influence and interest may vary with the decision at hand.

The salience stakeholder analysis framework provided a more comprehensive analysis considering the important stakeholder attributes. Since stakeholder engagement is relevant to effective healthcare priority setting, the framework used in this study and the study results are relevant to other healthcare priority setting processes in both high and low income countries. In Uganda, our analysis revealed politicians as salient stakeholders, these should be provided with information and “courted” to ensure that their priorities are aligned with the technocrat priorities. However, in order to strengthen democratic and equitable participation, concurrent efforts should focus on capacity strengthening for the latent and expectant stakeholders to enable then to meaningfully participate in- and influence healthcare priority setting and dealing with any potential power imbalances.

## CRediT authorship contribution statement

LK acquired the funding, conceptualized the study methodology, administered the project, participated in data collection, conceptualized the paper, contributed to the writting, reviewing and editing the manuscript. DSR: Participated in the data analysis and validation, writing the original draft, reviewing and editing the manuscript.

## Declaration of Competing Interest

The authors declare the following financial interests/personal relationships which may be considered as potential competing interests: This project was funded by the Canadian Institutes for Health Research (CIHR). The funding agency did not participate in any aspect of the project and manuscript development.
